# Is non-operative management safe and effective for all splenic blunt trauma? A systematic review

**DOI:** 10.1186/cc12868

**Published:** 2013-09-03

**Authors:** Roberto Cirocchi, Carlo Boselli, Alessia Corsi, Eriberto Farinella, Chiara Listorti, Stefano Trastulli, Claudio Renzi, Jacopo Desiderio, Alberto Santoro, Lucio Cagini, Amilcare Parisi, Adriano Redler, Giuseppe Noya, Abe Fingerhut

**Affiliations:** 1 Department of Digestive Surgery and Liver Unit, St. Maria Hospital, Viale Tristano di Joannuccio, 05100, Terni, Italy; 2 Department of General and Oncologic Surgery, University of Perugia, S. Andrea delle Fratte, 06156, Perugia, Italy; 3 Department of Surgery, Hillingdon Hospital, Pield Heath Road, Uxbridge, UB8 3NN; 4 Department of Surgical Sciences, Sapienza University of Rome, Policlinico Umberto Primo Sapienza, viale Regina Elena 324, 00161, Rome, Italy; 5 Department of Thoracic Surgery, University of Perugia, S. Andrea delle Fratte, 06156 Perugia, Italy; 6 First Department of Surgery, Hippocration Hospital, University of Athens Medical School, 75, M. Assias Street, Athens 115 27, Greece

## Abstract

**Introduction:**

The goal of non-operative management (NOM) for blunt splenic trauma (BST) is to preserve the spleen. The advantages of NOM for minor splenic trauma have been extensively reported, whereas its value for the more severe splenic injuries is still debated. The aim of this systematic review was to evaluate the available published evidence on NOM in patients with splenic trauma and to compare it with the operative management (OM) in terms of mortality, morbidity and duration of hospital stay.

**Methods:**

For this systematic review we followed the "Preferred Reporting Items for Systematic Reviews and Meta-analyses" statement. A systematic search was performed on PubMed for studies published from January 2000 to December 2011, without language restrictions, which compared NOM vs. OM for splenic trauma injuries and which at least 10 patients with BST.

**Results:**

We identified 21 non randomized studies: 1 Clinical Controlled Trial and 20 retrospective cohort studies analyzing a total of 16,940 patients with BST. NOM represents the gold standard treatment for minor splenic trauma and is associated with decreased mortality in severe splenic trauma (4.78% *vs*. 13.5% in NOM and OM, respectively), according to the literature. Of note, in BST treated operatively, concurrent injuries accounted for the higher mortality. In addition, it was not possible to determine post-treatment morbidity in major splenic trauma. The definition of hemodynamic stability varied greatly in the literature depending on the surgeon and the trauma team, representing a further bias. Moreover, data on the remaining analyzed outcomes (hospital stay, number of blood transfusions, abdominal abscesses, overwhelming post-splenectomy infection) were not reported in all included studies or were not comparable, precluding the possibility to perform a meaningful cumulative analysis and comparison.

**Conclusions:**

NOM of BST, preserving the spleen, is the treatment of choice for the American Association for the Surgery of Trauma grades I and II. Conclusions are more difficult to outline for higher grades of splenic injury, because of the substantial heterogeneity of expertise among different hospitals, and potentially inappropriate comparison groups.

## Introduction

Trauma is the fourth cause of death in the overall population and the first one in individuals below the age of 40 in Western countries [1,2]. Abdominal trauma can be classified as blunt or penetrating according to the agent and its mechanism of action [1-3].

The spleen is the most frequently injured organ in abdominal blunt trauma, mainly because of its highly vascularized parenchyma and its anatomic location. Spleen is the only structure involved in almost 46% of blunt trauma (BT). On the other hand the liver (41.7%), kidneys (16.4%), mesentery (15.1%), small and large bowel (10.1% and 6.3%, respectively), pancreas (5%) and omentum may concur with splenic injuries in the remaning part of BT [1,2].

Splenectomy was the only treatment proposed for splenic trauma until the 1960s. In 1968, Upadhyaya and Simpson proposed Non-Operative Management (NOM) in a study on 52 pediatric patients with splenic trauma [4].

The ultimate goal of NOM is to preserve the spleen and it is based on specific principles and criteria. NOM procedures include conservative medical treatment and angioembolization (AE) without access to the peritoneal cavity. Advantages of NOM for minor splenic trauma (grades I and II according to the American Association for the Surgery of Trauma-AAST), have been extensively reported, whereas its value for severe splenic injuries (AAST grades IV and V) is still under debate [2-4]. The aim of this systematic review was to evaluate the available published evidence on NOM in patients with splenic trauma and to compare it with operative management (OM) in terms of mortality, morbidity and duration of hospital stay.

## Methods

The criteria of the "Preferred Reporting Items for Systematic Reviews and Meta-analyses (PRISMA) statement" were followed in this systematic review [5].

### Inclusion criteria

We analyzed randomized controlled trials (RCTs) and non-randomized controlled studies (non-RCSs) comparing NOM *vs*. OM for blunt splenic trauma (BST). Only studies with at least 10 patients with BST were included. No language restrictions were imposed.

### Exclusion criteria

Studies were excluded if they involved only penetrating splenic trauma or if respective numbers of penetrating and blunt trauma were not specified.

### Participants

Participants were patients of all ages and either gender who had BST.

### Type of intervention

The intervention types were NOM (clinical observation, medical treatment and proximal or distal splenic angioembolization) *vs*. OM (total or partial splenectomy, splenorrhaphy, application of hemostatic agents).

### Sources of information

The following shows the systematic search performed on PubMed for papers published from January 2000 to December 2011:

- non[All Fields] AND operative[All Fields] AND ("therapy"[Subheading] OR "therapy"[All Fields] OR "treatment"[All Fields] OR "therapeutics"[MeSH Terms] OR "therapeutics"[All Fields]) AND ("spleen"[MeSH Terms] OR "spleen"[All Fields] OR "splenic"[All Fields])

- non[All Fields] AND operative[All Fields] AND ("organization and administration"[MeSH Terms] OR ("organization"[All Fields] AND "administration"[All Fields]) OR "organization and administration"[All Fields] OR "management"[All Fields] OR "disease management"[MeSH Terms] OR ("disease"[All Fields] AND "management"[All Fields]) OR "disease management"[All Fields]) AND ("spleen"[MeSH Terms] OR "spleen"[All Fields] OR "splenic"[All Fields]).

### Collection of data

We developed a data collection sheet, including names of authors, type of study, number of patients, type of treatment (NOM vs. OM) and the number of patients per treatment arm, mean age, Injury Severity Score (ISS), blood pressure, gender, American Association for the Surgery of Trauma (AAST) grade of splenic injury. Two authors (RC, ST) extracted data from included studies according to the data collection sheet, while another author (CB) oversaw the process. Controversies were solved by involving a fourth author (GN).

### Outcomes of interest

The primary endpoint of this systematic review was considered the overall mortality defined as any death that occurred after the start of NOM or OM and during the hospital stay.

Secondary endpoints were overall morbidity, overwhelming post-splenectomy infection (OPSI)/quality of life, blood transfusion, abdominal abscesses and length of hospital stay.

### Statistical analysis

Two authors performed the statistical analysis according to the PRISMA and the "Cochrane Handbook for Systematic Reviews" guidelines. Odds ratios (OR), (that is, the possibility that an event occurs in the two groups of treatment) were calculated for dichotomous outcomes while a weighted mean difference (WMD) was calculated to summarize the continuous outcomes. The Mantel-Haenszel method was used to combine OR for outcomes of interest. We also verified homogeneity among the studies by calculating the Chi² and the inconsistency (I²). As I² detected absence of homogeneity (>50%), the fixed effect model could not be used, and we, therefore, used the random effect model for analysis. Statistical analysis was conducted using the statistical software Review Manager Version 5.0 (The Nordic Cochrane Centre, The Cochrane Collaboration, 2008, Copenhagen, Denmark).

### Study selection

**Figure 1:**
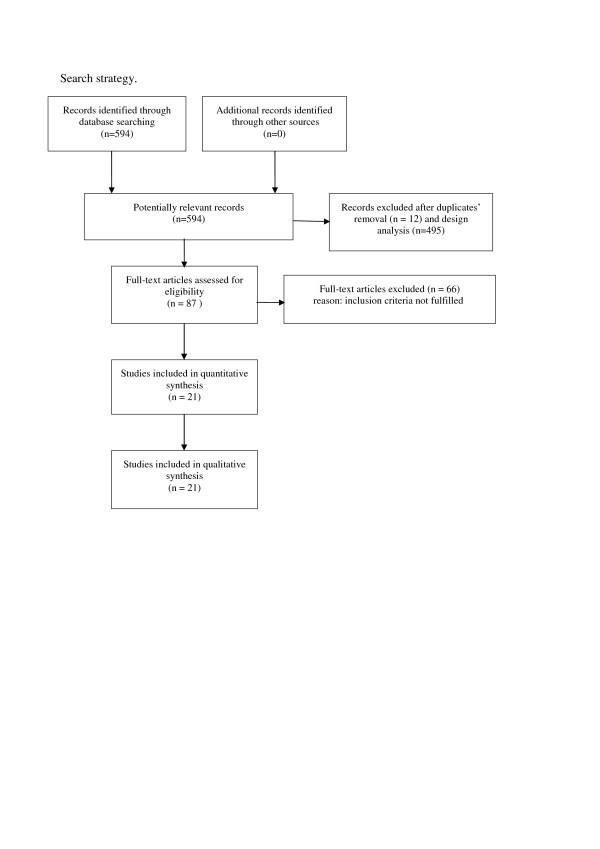
**Search strategy**.

Five hundred ninety-four studies were identified from our literature search. After review of titles and abstracts, 12 studies were excluded because of overlapping data and 495 because they were not relevant to the aims of our review. We analyzed the full texts of the 87 remaining studies, 21 of them met the inclusion criteria, while 66 were excluded (Figure 1).

### Search strategy results

We analyzed 21 non-randomized studies [1,6-25]: one clinical controlled trial and 20 retrospective cohort studies analyzing a total of 16,940 patients with blunt splenic injury (BSI); 12,449 underwent NOM *vs*. 4,491 OM (Additional file 1: Table S1).

### Quality of included studies

Quality of included studies was assessed by two authors (CR and ST) using the Newcastle-Ottawa Scale (NOS) [26] and is reported in Additional file 2: Table S2. All included studies had good methodological quality (>5 points) (mean 7.9 points, range 7 to 9).

### Risk of bias

#### Mean age

Twenty studies [1,6-14,16-25] reported participants' age: mean value was specified in 17 studies [1,6-10,12,14,16-25], the age range in one study [11] and median age in another study [13]. Crawford *et al*. reported the age of the 36 patients in whom treatment failed [15]. Mean age was 8.5 ± 5.2 years in the OM group and 9.2 ± 13 years in the NOM group in the study by Jim *et al. *[20] and 70.4 ± 9.3 in the group studied by Siriratsivawong *et al. *[16]. Only two studies [6,16] enrolled elderly patients, while in the other studies (patients' age was comprised between less than 1 [11] and 53 years [14]) (Additional file 3: Table S3).

Distribution of ages was not homogeneous, so it was not possible to compare studies according to this parameter.

#### Hemodynamic stability

Only four studies [8,10,18,21] reported hemodynamic characteristics of patients: Duchesne *et al*. and Dent *et al. *[8,18] defined patients as hemodynamically stable (HDS) if blood pressure was >100 mmHg. Scappellato *et al*. and Wahl *et al. *[10,21] treated HDS patients with NOM and hemodynamically unstable patients with OM. Blood pressure values were reported only in 10 studies [9-11,13,17,18,20-22,24], seven studies [9,10,16,18,20,22,24] reported the median systolic blood pressure, two studies [11,13] reported the number of patients with blood pressure above or below 90 mmHg and one study [21] reported the mean systolic/diastolic blood pressure. One study did not specify blood pressure criteria for NOM or OM [9]. Two studies [11,13] used the 90 mmHg cut-off without specifying how many patients had blood pressure between 90 and 100 mmHg; therefore, even hemodynamic unstable patients underwent NOM (Additional file 3: Table S3).

#### Injury Severity Score (ISS)

ISS was reported in 19 studies: mean value was reported in 3 studies [8,12,19], median value ± SD in 14 studies [1,6,7,9,10,14,16-18,20,22-25], the range of ISS was reported in 1 study [11], while in another study [13] ISS was defined with values above or below a cut-off of 25. It was possible to extrapolate the ISS value for the patients in the two compared groups (NOM *vs*. OM) from only one study [18], while Costa *et al. *[1], Claridge *et al. *[25], Gaarder *et al. *[14] and Harbrecht *et al. *[9] did not differentiate ISS values between NOM or OM. Cochran *et al. *[7] and Tsugawa *et al. *[6] defined ISS for pediatric or adult patients and for older patients, nevertheless without distinguishing between NOM and OM (Additional file 3: Table S3).

#### AAST

AAST grades of splenic lesions were reported in 11 studies: the number of patients relative to each AAST was reported in 6 studies [14,18,20-22], the mean AAST of NOM and OM groups were reported in 5 studies [6,10,16,23,24], and percentage of patients relative to AAST was reported in 1 study [25] (Additional file 3: Table S3).

#### Diagnostic protocol for BST

Thirteen studies reported a specific diagnostic algorithm for abdominal trauma management [1,10,11,13-18,21,23-25], while five studies [7,9,12,19,22] did not (Additional file 4: Table S4).

#### Imaging

Only one study [21] described the use of ultrasound, while others reported the use of only computed tomography (CT) of the abdomen (Additional file 4: Table S4).

## Results

### Overall mortality

Only three studies [18,20,22] reported the mortality relative to the AAST grading of splenic injuries. In particular, the mortality rate in patients with grades I and II of splenic injuries treated with NOM was 0%, but it was not possible to confirm the grading in patients with minor splenic injuries treated with OM since they did never required surgery [18,20,22]. The mortality in patients with grade III injuries was reported only by Duchesne *et al. *[18] and was similar in the two treatment groups (16% *vs*. 23% in NOM and OM, respectively). In patients with grades IV and V of splenic injuries the mortality was analyzed and reported in three studies [18,20,22]; mortality was lower in OM than in the NOM groups (mean rate 5.4% (45/337) *vs*. 13.3% (29/534), respectively). Data on the injuries of grade V was available only in the studies by Duchense *et al*., this was not possible from the studies by Jim *et al*. [20] and Velmahos *et al. *[22]. In fact, Duchesne *et al. *[18] distinguished mortality in grades IV and V (mean rate 31.5% *vs*. 13.7% in NOM and OM for grade IV - 12.5% *vs*. 66.6% in NOM and OM for grade V, respectively). Gaarder *et al. *[14] and Scappellato *et al. *[21], on the other hand, did not report the mortality with respect to the grade of lesion nor to treatment. (Additional file 5: Table S5).

### Overall morbidity

Only three studies [10,18,22] compared morbidity in the NOM and OM groups; six studies [6,8,12,14,15,21] reported data on morbidity but did not distinguish between the two treatment groups. In those three studies [10,18,22], it was possible to analyze morbidity according to the classification of Dindo-Clavien (DC) (Additional files 6, 7, 8: Tables S6-S8). Among 78 patients that underwent OM, 7 presented AAST grade III lesions. Of these, three had DC grade IV and four patients had DC grade V complications; in eight patients with AAST grade IV, two had DC grade IV and six had DC grade V complications. Among seven patients with AAST grade V, three had DC grade IV and four had DC grade V complications. Of the 76 patients who underwent NOM, 1 patient presented a AAST grade I lesion and a DC grade IV complication; 3 patients with AAST grade II had DC grade IV complications. Twelve patients had AAST grade III lesions, seven of whom had a DC grade IV while five had DC grade V complications. Among 15 patients with AAST grade IV lesions, 11 had DC grade IV and 4 had DC grade V complications. Among six patients with AAST grade V lesions, four had DC grade IV while two had DC grade V complications.

### Overwhelming post-splenectomy infection (OPSI)/quality of life

None of the included studies reported post-operative follow-up. For this reason, it was not possible to calculate the incidence of OPSI or quality of life after the treatment.

### Blood transfusion

Only seven studies [10,13-15,18,19,24] reported the number of blood transfusions (Additional file 9: Table S9). Only one study [24] reported the number of patients who received blood transfusions with respect to the type of treatment, specifying the number of patients who received more than one transfusion: 19/31 in the NOM group *vs*. 14/15 in the OM group.

### Abdominal abscesses

Only two studies [6,14] reported the incidence of abdominal abscesses, without specifying the method used for their detection. Furthermore, the authors did not specify in which group (NOM *vs*. OM) abscesses occurred and in how many cases they were associated with sepsis (Additional file 10: Table S10).

### Hospital stay

Duration of hospital stay was reported in 11 studies [6,7,9,12-14,16,18,20,22,25]. Only four studies [13,16,20,25] distinguished results between the two groups (NOM *vs*. OM). Claridge *et al. *[25] reported a median value ± SD (Additional file 11: Table S11).

The analysis of subgroups according to the DC classification and AAST grading for splenic trauma was possible only in the study by Duchesne *et al. *[18] (see Additional files 6 and 12, Tables S6 and S12). Among 76 patients that underwent NOM, 1 patient presented a AAST grade I lesion and was classed DC grade IV; in 2 patients, AAST grade II lesions were associated with DC grade IV complications. Twelve patients had AAST grade III lesions, seven of whom had DC grade IV while five had DC grade V complications. Among 15 patients with AAST grade IV lesions, 11 had DC grade IV and 4 had DC grade V complications. Among six patients with AAST grade V injury, four had DC grade IV and two had DC grade V complications. Among 78 patients that were treated with OM, 7 had AAST grade III injuries. Three of these patients had DC grade IV and four had DC grade V complications. Among eight patients with AAST grade IV lesions, two had DC grade IV and six had DC grade V complications. Among seven patients with AAST grade V, three had DC grade IV and four DC grade V complications. Overall, hospital stay in the NOM group was shorter than in OM, even if some studies reported a longer hospital stay in NOM because of more lasting monitoring and later return to daily activities [12,15].

## Discussion

In our systematic review, NOM represented the gold standard treatment for AAST grades I and II in 21 non-randomized studies (Additional file 1: Table S1) and was associated with decreased mortality in severe splenic trauma (AAST grades III to V) (4.78% *vs*. 13.5% in NOM and OM, respectively) (Additional file 5: Table S5) [20,22]. Mortality in OM group was higher, even if splenic bleeding was not always indicated as the single cause of death. In severe splenic trauma, it was not possible to determine post-treatment morbidity [10,18,22]. Furthermore, we could not establish which one of the treatments was more beneficial in terms of other analyzed outcomes (hospital stay and number of blood transfusions), since results either were not comparable among the studies or they were not reported [19,27-35].

In the past, the gold standard treatment for minor splenic lesions was early splenectomy [36-38], in order to avoid fatal hemorrhage [20,35,39]. Gradually, due to wider knowledge of the role and functions of the spleen, more surgeons preferred a conservative approach, either partial splenic salvage or NOM when possible [8,14,23,29,31,33,39-43]. NOM is a complex, multidisciplinary strategy that starts with careful clinical observation and constant strict monitoring by means of repeated laboratory tests and radiological imaging. Modern imaging techniques, such as multi-slice CT and contrast-enhanced ultrasound, have improved the quantitative definition of hemoperitoneum, evaluation of extension of splenic lesions, active bleeding and presence of concurring lesions in polytrauma patients [32,44-55].

At present, it appears that NOM can also be the first line treatment in some cases of severe splenic trauma (AAST grades III to V) when the decision between NOM and OM depends on careful risk-benefit analysis for each patient [10,31-33,56,57] as well as on the expertise of the surgeon and of the multidisciplinary team of the hospital.

Splenectomy is not exempt from intra-operative and post-operative complications, such as thrombocytosis, post-splenectomy infections, abdominal abscess and OPSI [59-61]. For these reasons, surgeons have preferred avoiding splenectomy. Nevertheless, the main risk of NOM is the possibility of sudden delayed hemorrhage that could be immediately fatal, before emergency surgery can be performed [1,8-11,14-17,20,22,24]. In addition, in NOM, the higher amounts of blood transfusion that are often required, thus increasing the risk of blood-borne disease, such as hepatitis [10,13-15,18,24,62], and the increased risk of not detecting other intra-abdominal lesions [17,18,39,63], have to be considered with respect to OM.

Unfortunately, follow-up was not reported in the studies included in this analysis; therefore, it was not possible to evaluate long-term complications [23,58,56]. The role and frequency of repeating imaging in the follow-up of patients treated with conservative treatment are still under debate [32,44-55]. Radiological imaging is necessary during the initial phases of NOM in order to evaluate eventual bleeding and abscess formation, and later, in order to detect the development of pseudo-aneurysms [18,32,44-55].

Clinical observation of the patient at the moment of hospitalization and monitoring of clinical conditions represent Ariadne's thread that brings caretakers through the labyrinth of laboratory and investigational tests and finally to success (or failure) of NOM [64-69].

## Conclusions

In conclusion, NOM has been accepted as standard treatment for AAST grades I and II BST, whereas this was not found to be safe in higher grades of splenic trauma. Currently, there is no consensus in the management of severe splenic trauma. Velmahos, a distinguished trauma surgeon [22], stated that "generalization about the overall success rates of NOM, should not represent severe blunt splenic injury".

Unfortunately, this review does not clarify the controversies regarding the safest therapeutic approach to severe splenic trauma (AAST grades IV and V) because of the selection bias in the recruitment of the NOM and OM groups, as well as missing data and heterogeneity of the studies included. Furthermore, all studies included in our review were retrospective and none were randomized due to the obvious difficulties in designing a randomized study on NOM of trauma patients.

Given the substantial heterogeneity between levels of expertise in the different hospitals, inclusion of patients with concurrent injuries of other organs and potentially inappropriate comparison groups the conclusions of the review may not be reliable for severe splenic trauma (AAST grades IV and V).

## Key messages

- NOM has been accepted as standard treatment for splenic trauma grades I and II.

- Advantages of NOM for the more severe splenic injuries are still debated.

- This review does not clarify the controversies regarding the safest treatment approach for severe splenic trauma because of the selection bias in the recruitment of the NOM and OM groups, as well as missing data and heterogeneity of the studies included.

- NOM can also be the initial treatment in some cases of severe splenic trauma when the decision between NOM and OM depends on careful risk-benefit analysis for each patient as well as on the expertise of the surgeon and of the multidisciplinary team of the hospital.

- The definition of hemodynamic stability varied greatly in the literature.

## Abbreviations

AAST: American Association for the Surgery of Trauma; AE: angioembolization; ATLS: Advanced Life Trauma Support; BSI: blunt splenic injury; BST: blunt splenic trauma; BT: blunt trauma; CCT: clinical control trial; CT: computed tomography; DC: the classification of Dindo-Clavien HDS: hemodynamically stable; ISS: Injury Severity Score; NOM: Non-Operative Management; NOS: Newcastle-Ottawa Scale; OM: operative management; OPSI: overwhelming post splenectomy infection; PRISMA: Preferred Reporting Items for Systematic Reviews and Meta-analyses statement; RCSs: randomized control studies; RCTs: randomized control trials

## Competing interest

The authors declare that they have no competing interests.

## Authors' contributions

All authors contributed equally to this work. In particular, RC conceived of the study. RC, CB, CL, AC, EF, ST, CR, JD, AS, LC, AP, AR, GN and AF participated in the original design. RC and ST extracted data from included studies according to the data extraction sheet, while CB oversaw the process. RC and EF analysed the data. RC and AF drafted the manuscript. All authors read and approved the final manuscript.

## Supplementary Material

Additional file 1 **Table S1**. Characteristics of included studies. Click here for file

Additional file 2 **Table S2**. Quality of included studied evaluated using the Newcastle-Ottawa Scale (NOS) for assessing the quality of non-randomized studies in meta-analyses. Click here for file

Additional file 3 **Table S3**. Patient characteristic in included studies. Click here for file

Additional file 4 **Table S4**. Diagnostic protocol for blunt splenic trauma (BST). Click here for file

Additional file 5 **Table S5**. Mortality of patients with respect to AAST grade of splenic lesion. Click here for file

Additional file 6 **Table S6**. Classification of morbidity according to Dindo-Clavien: NOM vs OM. Click here for file

Additional file 7 **Table S7**. Morbidity according to Dindo-Clavien classification: NOM vs OM. Click here for file

Additional file 8 **Table S8**. Morbidity according to Dindo-Clavien classification for AAST in NOM vs OM. Click here for file

Additional file 9 **Table S9**. Blood transfusions for different treatments (NOM vs OM). Click here for file

Additional file 10 **Table S10**. Abdominal abscesses in patients treated with NOM vs OM. Click here for file

Additional file 11 **Table S11**. Hospital stay after blunt splenic trauma: NOM vs OM. Click here for file

Additional file 12 **Table S12**. Morbidity according to Dindo-Clavien in blunt splenic trauma: NOM vs OM. Click here for file
