# Supplementary material for: Is non-operative management safe and effective for all splenic blunt trauma? A systematic review
Source: Crit Care. 2013 Sep 3;17(5):R185. doi: 10.1186/cc12868 (PMC4056798; doi:10.1186/cc12868)
Supplement: Additional file 1 — Table S1. Characteristics of included studies. [file cc12868-S1.DOCX]

Table 1: Characteristics of the included studies.

| Study | Type of study | Number of participants | | Treatment |
| --- | --- | --- | --- | --- |
|  |  |  |  |  |
| Tsugawa **[6]** | Multicentric retrospective cohort study | 117 pts with BST^1^ | 86 adults (< 60 yrs) | 32 OM^2^ (25 splenectomies, 7 splenorrhaphies) |
|  |  |  |  | 54 NOM^3^ |
|  |  |  | 31 patients > 60 yrs | 15 OM (10 splenectomies, 5 splenorrhaphies) |
|  |  |  |  | 16 NOM |
| Cochran **[7]** | Multicentric retrospective cohort study | 159 pediatric pts with BSI | | 31 OM |
|  |  |  |  | 128 NOM |
|  |  | 305 adult pts with BSI | | 470 OM |
|  |  |  |  | 165 NOM |
| Dent **[8]** | Monocentric retrospective cohort study | 168 pts HDS^4^ | 28 OM | 24 splenectomies 4 splenorrhaphies |
|  |  |  | 140 NOM | 8 SAE^5^ with 1 failure that required splenectomy |
|  |  |  |  | 132 pts under observation, 2 failures that required splenectomy and 5 SAE |
| Harbrecht **[9]** | Multicentric retrospective cohort study | 2,138 pts with BSI  (1,366 level 1, 772 level 2) | | OM: 419 level 1, 295 level 2 |
|  |  |  |  | NOM: 947 level 1 (with 167 failures), 477 level 2 (with 62 failures) |
| Wahl **[10]** | Monocentric retrospective cohort study | 164 pts  with BST | 11 hemodynamically  unstable pts | 33 OM; 4splenorrhaphies and 29 splenectomies |
|  |  |  | 153 pts HDS | 131NOM: 24 SAE, |
|  |  |  |  | 107 pts under observation, 3 failures treated with splenectomy |
| McIntyre **[11]** | Multicentric retrospective cohort study | 2,243 pts  with BST | | 610 OM  (484 splenectomies,  17 partial splenectomies, 106 splenorrhaphies) |
|  |  |  |  | 1,633 NOM (SAE, clinical observation and bed rest, repeated laboratory tests)  with 252 failures (194 splenectomies, 4 partial splenectomies, 50 splenorrhaphies, 4 SAE) |
| Mooney **[12]** | Multicentric retrospective cohort study | 2,191 pts with BST | | 337 OM (253 splenectomies and 84 splenorrhaphies) |
|  |  |  |  | 1,854 NOM |
| Cadeddu **[13]** | Monocentric retrospective cohort study | 266 pts with BST | | 118 OM |
|  |  |  |  | 148 NOM |
| Gaarder **[14]** | Monocentric retrospective cohort study | 133 pts  with BST | Group 1:  69 pts with BSI  before the systematic introduction of SAE in the protocol | 30 OM (total splenectomies) |
|  |  |  |  | 39 NOM with 8 failures (5 splenectomies, 1 splenorrhaphy and two non therapeutic laparotomies) |
|  |  |  | Group 2:  64 pts with BSI  after the systematic introduction of SAE in the protocol | 16 OM (total splenectomies) |
|  |  |  |  | 47 NOM with 2 failures (total splenectomy) |
| Crawford **[15]** | Monocentric retrospective cohort study | 691 pts  with BST | | 192 OM (168 splenectomies, 24 splenorrhaphies) |
|  |  |  |  | 499 NOM with 36 failures (26 early failure and 10 delayed, treated with splenectomy) |
| Siriratsivawong  **[16]** | Multicentric retrospective cohort study | 1,008 pts  with BST  (≥ 55 yrs) | | 402 OM |
|  |  |  |  | 606 NOM with 151 failures |
|  |  |  |  |  |
| Harbrecht **[17]** | Monocentric retrospective cohort study | 570 pts with BST | | 221 OM |
|  |  |  |  | 349 NOM:  46 SAE, with 32 failures |
| Duchesne **[18]** | Monocentric retrospective cohort study | 154 HDS pts with  computed tomographic (CT) evidence of active contrast extravasation | | 78 pts underwent  surgery (splenectomy) |
|  |  |  |  | 76 pts treated with proximal SAE |
| Bowman **[19]** | Multicentric retrospective cohort study | 5,061pts  with BST | | 756 underwent OM  (total splenectomy) |
|  |  |  |  | 4305 NOM |
| Jim **[20]** | Monocentric retrospective cohort study | 413 pts  with severe BST | | 128 OM |
|  |  |  |  | 285 NOM  With 45 failures (36 total splenectomies, 6 splenorrhaphies, 3 “no splenic procedure”) |
| Scappellato **[21]** | Monocentric retrospective cohort study | 56 | 27 pts hemodynamically  unstable | 29 pts underwent surgery (splenectomy) |
|  |  |  | 29 pts HDS | 27 NOM |
| Velmahos **[22]** | Multicentric retrospective cohort study | 388 with BST | BST grade IV:  314 pts | 164 OM |
|  |  |  | BST grade V:  74 pts | 224 NOM:  85 failures required splenectomy (34.5% in pts with grade IV and 60% in pts with grade V). |
| Costa **[1]** | Monocentric retrospective cohort study | 36 pts  with BST | 22 “major splenic rupture” | 22 OM :  (21 splenectomies and one “laparoscopic procedure to control bleeding” |
|  |  |  | 10 contusions/ hematomas | 14 NOM (SAE):  1 failure: splenectomy 8 days after SAE |
|  |  |  | 4 minor splenic tearings |  |
| Malhotra **[23]** | Prospective study CCT | 12 pts with BST, hemodynamic stability is not reported | | OM: 4 splenectomies |
|  |  |  |  | NOM: 8 underwent SAE |
| Bruce **[24]** | Multicentric retrospective cohort study | 236 pts  with isolated BST | | 195 pts under observation:  5 failures with one SAE and 4 splenectomies |
|  |  |  |  | 11 splenectomies or splenorrhaphies with one reintervention |
|  |  |  |  | 30 underwent SAE with 3 failures (splenectomies) |
| Claridge **[25]** | Monocentric retrospective cohort study | 431 pts with BST | | 55 OM |
|  |  |  |  | 376 NOM |

^1^ Blunt Splenic Trauma

^2^ Operative Management

^3^ Non Operative Management

^4^ Hemodinamically stable

^5^ Splenic Angioembolization
