# Supplementary material for: Is non-operative management safe and effective for all splenic blunt trauma? A systematic review
Source: Crit Care. 2013 Sep 3;17(5):R185. doi: 10.1186/cc12868 (PMC4056798; doi:10.1186/cc12868)
Supplement: Additional file 2 — Table S2. Quality of included studied evaluated using the Newcastle-Ottawa Scale (NOS) for assessing the quality of non-randomized studies in meta-analyses. [file cc12868-S2.DOCX]

Table 2: Quality of included studied evaluated using Newcastle-Ottawa Scale (NOS) for assessing the quality of non randomized studies in meta-analyses.

| Author | Year | Selection | | | | Compatibility | Outcomes | | | Tot. |
| --- | --- | --- | --- | --- | --- | --- | --- | --- | --- | --- |
|  |  | 1 | 2 | 3 | 4 | 5 | 6 | 7 | 8 |  |
| Tsugawa **[6]** | 2002 | * | * | * | - | ** | * | * | * | 8 |
| Cochran **[7]** | 2004 | * | * | * | - | ** | * | * | * | 8 |
| Dent **[8]** | 2004 | * | * | * | - | * | * | * | * | 7 |
| Harbrecht **[9]** | 2004 | * | * | * | - | ** | * | * | * | 8 |
| Wahl **[10]** | 2004 | * | * | * | - | ** | * | * | * | 8 |
| McIntyre **[11]** | 2005 | * | * | * | - | * | * | * | * | 7 |
| Mooney **[12]** | 2006 | * | * | * | * | ** | * | * | * | 9 |
| Cadeddu **[13]** | 2006 | * | * | * | - | ** | * | * | * | 8 |
| Gaarder **[14]** | 2006 | * | * | * | - | ** | * | * | * | 8 |
| Crawford **[15]** | 2007 | * | * | * | - | ** | * | * | * | 8 |
| Siriratsivawong **[16]** | 2007 | * | * | * | - | ** | * | * | * | 8 |
| Harbrecht **[17]** | 2007 | * | * | * | - | * | * | * | * | 7 |
| Duchesne **[18]** | 2008 | * | * | * | - | ** | * | * | * | 8 |
| Bowman **[19]** | 2008 | * | * | * | * | ** | * | * | * | 9 |
| Jim **[20]** | 2008 | * | * | * | - | ** | * | * | * | 8 |
| Scappellato **[21]** | 2009 | * | * | * | - | * | * | * | * | 7 |
| Velmahos **[22]** | 2010 | * | * | * | - | ** | * | * | * | 8 |
| Costa **[1]** | 2010 | * | * | * | - | * | * | * | * | 7 |
| Malhotra **[23]** | 2010 | * | * | * | - | * | * | * | * | 7 |
| Bruce **[24]** | 2011 | * | * | * | * | ** | * | * | * | 9 |
| Claridge **[25]** | 2011 | * | * | * | * | ** | * | * | * | 9 |

*one point; **two points
