# Supplementary material for: Is non-operative management safe and effective for all splenic blunt trauma? A systematic review
Source: Crit Care. 2013 Sep 3;17(5):R185. doi: 10.1186/cc12868 (PMC4056798; doi:10.1186/cc12868)
Supplement: Additional file 3 — Table S3. Patient characteristic in included studies. [file cc12868-S3.DOCX]

Table 3: Patient characteristics of patients in included studies.

| Study | Age | ISS^1^ | PA  (mmHg) | Sex | AAST ^2^ | | | | | |
| --- | --- | --- | --- | --- | --- | --- | --- | --- | --- | --- |
|  |  |  |  |  | Grade | I | II | III | IV | V |
| Tsugawa **[6]** | 42.5^3^±18.5SD^4^ | 21.5^5^±14.5SD | nr | nr | Mean grade between young patients 2.9 ± 1.5 | | | | | |
|  |  |  |  |  |  |  |  |  |  |  |
|  | 68.5^3^±8.5SD | 28.5^5^±12.5SD | nr | nr | Mean grade between old patients 3.3 ± 1.4 | | | | | |
|  |  |  |  |  |  |  |  |  |  |  |
| Cochran **[7]** | 12.3^3^±4.2SD | 16.1^5^±12.4SD | nr | 105 M  54 F | OM^6^ pts | nr | nr | nr | nr | nr |
|  |  |  |  |  |  |  |  |  |  |  |
|  | 35.9^3^±17.4SD | 21.8^5^±12.7SD | nr | 198 M  107 F | NOM^7^ pts | nr | nr | nr | nr | nr |
|  |  |  |  |  |  |  |  |  |  |  |
| Dent **[8]** | 33.4^3^ | 19.6^3^ | nr | nr | OM pts | nr | nr | nr | nr | nr |
|  |  |  |  |  | NOM pts | Mean 2.7 | | | | |
|  |  |  |  |  |  |  |  |  |  |  |
| Harbrecht **[9]** | 37.8^3^± 0.4SD | 26.1^5^±0.3SD | 122^5^±1SD | 1,402 M;  736  F | OM pts | nr | nr | nr | nr | nr |
|  |  |  |  |  | NOM pts | nr | nr | nr | nr | nr |
| Wahl **[10]** | 37^3^±15SD | 35^5^±12SD | 105^5^±28SD | 25 M 8 F | OM pts | Mean AIS^8^ 3.4 ± 0.9 | | | | |
|  | 50^3^±20SD | 27^5^±11SD | 121^5^±26SD | 17 M  7 F | NOM pts | Mean AIS for SAE^9^ 3.8 ± 0.4  Mean AIS for pts under observation 2.4 ± 0.7 | | | | |
|  | 38^3^±18SD | 22^5^±13SD | nr | 74 M  33 F |  |  |  |  |  |  |
| McIntyre **[11]** | 49 pts  0-13;  488 pts  14-55;  73 pts  > 55 | 41pts  1-8 yrs;  83pts  9-16 yrs;  162 pts  17-25 yrs;  324 pts  > 25 yrs | 119 pts  < 90  440 pts >90 | nr | OM pts  NOM pts | nr | nr | nr | nr | nr |
|  | 284 pts  0-13;  956 pts  14-55;  141 pts  ≥56 | 381pts  1-8  332pts  9-16  347pts  17-25  321pts  ≥26 | 89pts  < 90  990pts  ≥ 90 | nr |  |  |  |  |  |  |
| Mooney **[12]** | 292 pts <6  666pts  6-10  1,233pts  >10 | 9^3^ | nr | 1,615  M;  576 F | OM pts | nr | nr | nr | nr | nr |
|  |  |  | nr |  | NOM pts | nr | nr | nr | nr | nr |
| Cadeddu **[13]** | 37^5^ | 93 pts > 25 | 15pts systolic  < 90 | 84 M  34 F | OM pts | nr | nr | nr | nr | nr |
|  | 39^5^ | 60 pts > 25 | 10 pts  systolic < 90 | 99 M  49 F | NOM pts | nr | nr | nr | nr | nr |
| Gaarder **[14]** | 33.8^3^ | 31.1^5^±17.8SD | nr | 55 M  14 F | OM pts | 52 pts | | | 14 pts | |
|  |  |  |  |  | NOM pts |  |  |  | 3 pts(2 failure) | |
|  | 34.5^3^ | 30^5^±12.6SD | nr | 46 M  18 F | OM pts | 41 pts | | | 8 pts | |
|  |  |  |  |  | NOM pts |  |  |  | 15 pts (1 failure) | |
| Crawford **[15]** | Only data relative to 36 failures are reported in order to evaluate possible causes of late complications | | | | OM pts | nr | nr | nr | nr | nr |
|  |  |  |  |  | NOM pts | nr | nr | nr | nr | nr |
| Siriratsivawong  **[16]** | 70.4^3^±9.3SD | 33.5^5^±14.6SD | nr | 523M  485 F | OM pts | Mean grade 3.46 ± 0.06 | | | | |
|  |  | 21.7^5^±12.4SD | nr |  | NOM pts | sNOM^10^ mean grade 2.38 ± 0.03 | | | | |
|  |  | 27.1^5^±10.8SD |  |  |  | fNOM^11^ mean grade 3.25 ± 0.09 | | | | |
| Harbrecht **[17]** | 42.1^3^±1.4SD | 30.7^5^±1SD | SBP^12^  104^5^±3SD | nr | OM pts | nr | nr | nr | nr | nr |
|  | 38.4^3^±1SD | 21^5^±0.6SD | SBP  130^5^±1SD | nr | NOM pts | nr | nr | nr | nr | nr |
| Duchesne **[18]** | 33^3^±14SD | 31^5^±13SD | 132^5^±29SD | 31 M  47 F | OM pts | 5 | 7 | 21 | 29 | 16 |
|  | 37^3^±17SD | 29^5^±11SD | 119^5^±24SD | 24 M  47 F | NOM pts (SAE) | 10 | 16 | 25 | 19 | 6 |
| Bowman **[19]** | 15.1^3^ | 26^3^ | nr | 553M  203F | OM pts | nr | nr | nr | nr | nr |
|  | 13.1^3^ | 13.9^3^ | nr | 3,076M  1,229F | NOM pts | nr | nr | nr | nr | nr |
| Jim **[20]** | 8.5^3^±5.2SD | 35^5^±15SD | 108^5^±33SD | 97 M  31 F | OM pts | 0 | 0 | 0 | 128 | |
|  | 9.2^3^±4.2SD | 27^5^±13SD | 115^5^±25SD | 208 M  77 F | NOM pts | 0 | 0 | 0 | 285 | |
| Scappellato **[21]** | 34.6^3^ | nr | 112/64^3^ | 24 M  5 F | OM pts | 0 | 5 | 11 | 10 | 3 |
|  | 37.2^3^ | nr | 117/71^3^ | 23 M  4 F | NOM pts (SAE) | 7 | 10 | 6 | 4 | 0 |
| Velmahos **[22]** | 38^3^±17SD | 36^5^±13SD | systolic  109^5^±27SD mmHg | 114 M  50 F | OM pts | 0 | 0 | 0 | 121 | 43 |
|  | 39^3^±17SD | 27^5^±11SD | systolic  121^5^±26SD mmHg | 155 M  69 F | NOM pts | 0 | 0 | 0 | 193 | 31 |
| Costa **[1]** | 38.7^3^±16.2SD | 31.1^3^±14.4SD | nr | 83.6% M;  16.4% F | OM pts | nr | | | | |
|  |  |  | nr |  | NOM pts | nr | | | | |
|  |  |  |  |  |  |  |  |  |  |  |
| Malhotra **[23]** | 34^3^±5.1SD | 21^5^±5.2SD | nr | 3 M  1 F | OM pts | Mean Grade  3.5 ± 0.6 | | | | |
|  | 34^3^±6.1SD | 25^5^±4.3SD | nr | 4 M  4 F | NOM pts | Mean Grade  3.6 ± 0.5 | | | | |
| Bruce **[24]** | 31.2^3^±18.5SD | 14.6^5^±7.2SD | 123.6^5^±22.7SD | 135 M  60 F | Pts under observation | 2.5 ± 1 | | | | |
|  | 53.8^3^±19.7SD | 18.5^5^±6.7SD | 107.4^5^±46.3SD | 7 M  4 F | OM pts | 3.9 ± 9 | | | | |
|  | 39^3^±19.9SD | 15.5^5^±6.6SD | 126^5^±20SD | 22 M  8 F | NOM pts | 3.6 ± 7 | | | | |
| Claridge **[25]** | 34^3^±1SD | 21^5^±1SD | nr | 67%M  33% F | OM pts | 12% | 35% | 26% | 23% | 4% |
|  |  |  | nr |  | NOM pts |  |  |  |  |  |

^1^ Injury severity score

^2^ classification of the American Association for the Surgery of Trauma

^3^Mean

^4^Standard deviation

^5^Median

^6^Operative Management

^7^Non Operative Management

^8^Abbreviated Injury Scale

^9^Splenic Angioembolization

^10^ successful NOM

^11^failure of NOM

^12^Systolic blood pressure
