# Supplementary material for: Is non-operative management safe and effective for all splenic blunt trauma? A systematic review
Source: Crit Care. 2013 Sep 3;17(5):R185. doi: 10.1186/cc12868 (PMC4056798; doi:10.1186/cc12868)
Supplement: Additional file 4 — Table S4. Diagnostic protocol for blunt splenic trauma (BST). [file cc12868-S4.DOCX]

Table 4: Diagnostic protocol for BST

| Author | Hosptital | Years | | Diagnostic protocol | Ultrasound | CT or  Angio CT |
| --- | --- | --- | --- | --- | --- | --- |
| Tsugawa **[6]** | 2 faculties of Medicine | 1983-1997 | | no | no | CT mdc |
| Cochran **[7]** | 2 level 1 trauma centre | 1996-1997 | | nr^1^ | nr | nr |
|  | Regional Pediatric referral centre |  |  |  |  |  |
| Dent **[8]** | Level 1 trauma centre | 1998-2000 | | no | no | CT mdc  Angio CT |
| Harbrecht **[9]** | 15 level 1 trauma centre | 1998-2000 | | nr | nr | nr |
|  | 12 level 2 trauma centre |  |  |  |  |  |
| Wahl **[10]** | Level 1 trauma centre | 2000-2003 | | yes | no | CT mdc |
| McIntyre **[11]** | Level 1 trauma centre | 1995-2001 | | yes | nr | nr |
| Mooney **[12]** | Data extracted from database of pediatric patients of 2000 | 2000 | | nr | nr | nr |
| Cadeddu **[13]** | Tertiary trauma centre | 1992-2001 | | yes | no | CT mdc |
| Gaarder **[14]** | University Hospital | 2000-2004 | 2000-2002 | no | no | CT |
|  |  |  | 2002-2004 | yes | no | CT  Angio CT |
| Crawford **[15]** | level 1 trauma centre | 1993-2005 | | yes | no | CT mdc |
| Siriratsivawong  **[16]** | 27 trauma centers | 1993-2001 | | yes | nr | nr |
| Harbrecht **[17]** | Pittsburgh Medical Centre | 200-2004 | | yes | no | Angio CT |
| Duchesne **[18]** | Level 1 trauma centre | 2000-2005 | | yes | no | CT con mdc |
| Bowman **[19]** | Database of pediatric patients of 2000 and2003 | 2000 and 2003 | | nr | nr | nr |
| Jim **[20]** | School of medicine Los Angeles | 2001-2005 | | no | nr | nr |
| Scappellato **[21]** | Vittorio Emanuele Hospital of Catania | 2000-2005 | | yes | yes | CT |
| Velmahos **[22]** | 11 level 1 trauma centre | 2001-2008 | | nr | no | CT |
|  | 3 level 2 trauma centre |  |  |  |  |  |
| Costa **[1]** | Hospital of S.Andrea of Roma | 2006-2007 | | yes | nr | nr |
| Malhotra **[23]** | Level 1 trauma centre | nr | | yes | no | Angio CT |
| Bruce **[24]** | 2 level 1 trauma centre | 2005-2009 | | yes | no | Angio CT  CT con mdc |
| Claridge **[25]** | Level 1 trauma centre | 2003-2009 | | yes | no | CT con mdc |

^1^not reported
