# Supplementary material for: Is non-operative management safe and effective for all splenic blunt trauma? A systematic review
Source: Crit Care. 2013 Sep 3;17(5):R185. doi: 10.1186/cc12868 (PMC4056798; doi:10.1186/cc12868)
Supplement: Additional file 5 — Table S5. Mortality of patients with respect to AAST grade of splenic lesion. [file cc12868-S5.DOCX]

Table 5: Mortality of patients with respect to AAST grading of splenic lesion.

| Study | Total number  of patients | Type of treatment | AAST^1^ | | | | |
| --- | --- | --- | --- | --- | --- | --- | --- |
|  |  |  | I | II | III | IV | V |
| Duchesne **[18]** | 78 | OM^2^ | 0 | 0 | 4 | 6 | 4 |
|  | 76 | NOM^3^ | 0 | 0 | 5 | 4 | 2 |
| Jim **[20]** | 128 | OM | 0 | 0 | 0 | 12 | |
|  | 285 | NOM | 0 | 0 | 0 | 17 | |
| Velmahos **[22]** | 164 | OM | 0 | | | 28 | |
|  | 224 | NOM | 0 | | | 5 | |
| Total | 370 | OM | 5 | | | 50 | |
|  | 585 | NOM | 4 | | | 28 | |

^1^ classification of the American Association for the Surgery of Trauma

^2^operative management

^3^non operative management
