# Supplementary material for: Is non-operative management safe and effective for all splenic blunt trauma? A systematic review
Source: Crit Care. 2013 Sep 3;17(5):R185. doi: 10.1186/cc12868 (PMC4056798; doi:10.1186/cc12868)
Supplement: Additional file 6 — Table S6. Classification of morbidity according to Dindo-Clavien: NOM vs OM. [file cc12868-S6.DOCX]

Table 7: Morbidity according to Dindo-Clavien classification: NOM vs OM.

| Study | Total number of patients | Treatment | Morbidity according to Dindo-Clavien classification | | | | |
| --- | --- | --- | --- | --- | --- | --- | --- |
|  |  |  | I | II | III | IV | V |
| Tsugawa **[6]**  117 pts |  | OM^2^ | 65 | 0 | 5.22±3.51 young pts;  6.77±3.15 old pts | 0 | 21 |
|  |  | NOM^3^ |  |  |  |  |  |
| Cochran **[7]**  464 pts |  | OM | 0 | 0 | Nr | nr^4^ | nr |
|  |  | NOM |  |  |  |  | nr |
| Dent **[8]** | 28 | OM | 0 | 0 | 0 | 1  IV b | nr |
|  | 140 | NOM |  |  |  |  |  |
| Harbrecht **[9]** | 714 | OM | nr | nr | Nr | nr | nr |
|  | 1424 | NOM | nr | nr | nr | nr | nr |
| Wahl **[10]** | 36 | OM | 0 | 0 | 9 | 10 | 8 |
|  | 238 | NOM | 0 | 4 | 1 | 2 | 2 |
| McIntyre **[11]** | 610 | OM | nr | nr | nr | nr | nr |
|  | 1633 | NOM | nr | nr | nr | nr | nr |
| Mooney **[12]** | 337 | OM | nr | nr | nr | 406 | nr |
|  | 1850 | NOM |  |  |  |  |  |
| Cadeddu **[13]** | 118 | OM | nr | nr | nr | nr | nr |
|  | 148 | NOM | nr | nr | nr | nr | nr |
| Gaarder **[14]**  133 pts |  | OM | 0 | 0 | 102 | 26 | 17 |
|  |  | NOM |  |  |  |  |  |
| Crawford **[15]** | 228 | OM | 0 | 0 | 27 | 0 | 0 |
|  | 463 | NOM |  |  |  |  |  |
| Siriratsivawong  **[16]** | 402 | OM | nr | nr | nr | nr | nr |
|  | 455 | NOM | nr | nr | nr | nr | nr |
| Harbrecht **[17]** | 221 | OM | nr | nr | nr | nr | nr |
|  | 349 | NOM | nr | nr | nr | nr | nr |
| Duchesne **[18]** | 78 | OM | 0 | 0 | nr | 8 | 14 |
|  | 76 | NOM | 0 | 0 | nr | 26 | 11 |
| Bowman **[19]** | 756 | OM | nr | nr | nr | nr | nr |
|  | 4305 | NOM |  |  |  |  |  |
| Jim **[20]** | 128 | OM | nr | nr | nr | nr | nr |
|  | 1633 | NOM | nr | nr | nr | nr | nr |
| Scappellato **[21]** | 29 | OM | 0 | 0 | 6 | 0 | 3 |
|  | 27 | NOM |  |  |  |  |  |
| Velmahos **[22]** | 249 | OM | nr | nr | 24 | 28 | 28 |
|  | 139 | NOM | nr | nr | 58 | 22 | 5 |
| Costa **[1]** | 22 | OM | nr | nr | nr | nr | nr |
|  | 14 | NOM | nr | nr | nr | nr | nr |
| Malhotra **[23]** | 4 | OM | nr | nr | nr | nr | nr |
|  | 8 | NOM | nr | nr | nr | nr | nr |
| Bruce **[24]** | 15 | OM | nr | nr | nr | nr | nr |
|  | 221 | NOM | nr | nr | nr | nr | nr |
| Claridge **[25]** | 55 | OM | nr | nr | nr | nr | nr |
|  | 376 | NOM | nr | nr | nr | nr | nr |
| Total | 4030 | OM | nr | nr | 33* | 46* | 50* |
|  | 13499 | NOM | nr | 4* | 59* | 50* | 18* |

^1^ classification of the American Association for the Surgery of Trauma

^2^operative management

^3^non operative management

^4^ not reported

* Data that are included in the total number come from only studies that distinguished patients treated with NOM and OM
