# Supplementary material for: Is non-operative management safe and effective for all splenic blunt trauma? A systematic review
Source: Crit Care. 2013 Sep 3;17(5):R185. doi: 10.1186/cc12868 (PMC4056798; doi:10.1186/cc12868)
Supplement: Additional file 7 — Table S7. Morbidity according to Dindo-Clavien classification: NOM vs OM. [file cc12868-S7.DOCX]

Table 8: Morbidity according to Dindo-Clavien classification for AAST in NOM vs OM.

|  | | Morbidity according to Dindo-Clavien classification | | | | | |
| --- | --- | --- | --- | --- | --- | --- | --- |
| Study | Tot.  Number of patients | Treatment | I | II | III | IV | V |
| Wahl [10] | 36 | OM^1^ | 0 | 0 | 9 | 10 | 8 |
|  | 238 | NOM^2^ | 0 | 4 | 1 | 2 | 2 |
| Duchesne [18] | 78 | OM | 0 | 0 | nr^3^ | 8 | 14 |
|  | 76 | NOM | 0 | 0 | nr | 26 | 11 |
| Velmahos [22] | 249 | OM | nr | nr | 24 | 28 | 28 |
|  | 139 | NOM | nr | nr | 58 | 22 | 5 |
| Total | 363 | OM | 0 | 0 | 33 | 46 | 50 |
|  | 453 | NOM | 0 | 4 | 59 | 50 | 18 |

^1^operative management

^2^non operative management

^3^not reported
