# Supplementary material for: Is non-operative management safe and effective for all splenic blunt trauma? A systematic review
Source: Crit Care. 2013 Sep 3;17(5):R185. doi: 10.1186/cc12868 (PMC4056798; doi:10.1186/cc12868)
Supplement: Additional file 8 — Table S8. Morbidity according to Dindo-Clavien classification for AAST in NOM vs OM. [file cc12868-S8.DOCX]

Table 9: Blood transfusions according to the treatment.

| Author | Blood Transfusion [units] | Blood loss [cc/Kg] |
| --- | --- | --- |
| Tsugawa **[6]** | nr^1^ | Young patients: 38.5±16.9 in patients that underwent splenectomy, 20.5±21.5 in NOM^2^ or splenorrhaphy.  Old patients:43.5±19.8 in patients that underwent splenectomy,25.5±23.4 in NOM or splenorrhaphy. |
| Cochran **[7]** | nr | nr |
| Dent **[8]** | nr | nr |
| Harbrecht **[9]** | nr | nr |
| Wahl **[10]** | 3.4±2.9 for OM^3^, 1.5±1.7 for NOM (mean±SD) | nr |
| McIntyre **[11]** | nr | nr |
| Mooney **[12]** | nr | nr |
| Cadeddu **[13]** | 6 for OM and 1 for NOM (median) | nr |
| Gaarder **[14]** | 7.6 in group 1 and 6 in group 2 | nr |
| Crawford **[15]** | Only data relative to 10 delayed failures of treatment are reported | nr |
| Siriratsivawong **[16]** | nr | nr |
| Harbrecht **[17]** | nr | nr |
| Duchesne **[18]** | OM: 4.4 in grade I;  5 in grade II;  8.2 in grade III; 4.6 in grade IV; 3.6 in grade V. | nr |
|  | NOM: 4.5 in grade I; 9.6 in grade II; 9.9 in grade III; 11.7 in grade IV; 4 in grade V |  |
| Bowman **[19]** | nr | nr |
| Jim **[20]** | nr | nr |
| Scappellato **[21]** | nr | nr |
| Velmahos **[22]** | nr | nr |
| Costa **[1]** | nr | nr |
| Malhotra **[23]** | nr | nr |
| Bruce **[24]** | 14 for OM, 19 for NOM | nr |
| Claridge **[25]** | nr | nr |

^1^not reported

^2^non operative management

^3^operative management
