# Supplementary material for: Is non-operative management safe and effective for all splenic blunt trauma? A systematic review
Source: Crit Care. 2013 Sep 3;17(5):R185. doi: 10.1186/cc12868 (PMC4056798; doi:10.1186/cc12868)
Supplement: Additional file 9 — Table S9. Blood transfusions for different treatments (NOM vs OM). [file cc12868-S9.DOCX]

Table 10: Abdominal abscesses according to the treatment.

| Study | Tot.  Pts | Treatment | AAST ^1^ | | | | |
| --- | --- | --- | --- | --- | --- | --- | --- |
|  |  |  | I | II | III | IV | V |
| Tsugawa **[6]** | 117 | OM^2^ | There is a distinction for age and not for grade:  3 in young patients, 2 in old patients | | | | |
|  |  | NOM^3^ |  |  |  |  |  |
| Gaarder **[14]** | 133 | OM | There is a distinction for group and not for grade:  5 patients in group 1, 2 patients in group 2 | | | | |
|  |  | NOM |  |  |  |  |  |
| Duchesne **[18]** | 78 | OM | nr^4^ | | | | |
|  | 76 | NOM | nr | | | | |

^1^ classification of the American Association for the Surgery of Trauma

^2^ operative management

^3^ non operative management

^4^ not reported
