# Supplementary material for: Is non-operative management safe and effective for all splenic blunt trauma? A systematic review
Source: Crit Care. 2013 Sep 3;17(5):R185. doi: 10.1186/cc12868 (PMC4056798; doi:10.1186/cc12868)
Supplement: Additional file 10 — Table S10. Abdominal abscesses in patients treated with NOM vs OM. [file cc12868-S10.DOCX]

Table 11: Hospital stay after splenic trauma: NOM vs OM

| Author | Hospital stay [days] |
| --- | --- |
| Tsugawa **[6]** | 18.5^1^±7.5SD^2^for young patients and 26.5^1^±8.1SD for old patients |
| Cochran **[7]** | 5^1^ for pediatric patients, 8-6^1^ for adults |
| Dent **[8]** | Nr^3^ |
| Harbrecht **[9]** | 12^1^±0.4SD in level I trauma centers and 10.1^1^±0.4SD in level II |
| Wahl **[10]** | Nr |
| McIntyre **[11]** | Nr |
| Mooney **[12]** | 4^1^ |
| Cadeddu **[13]** | 21^1^ for NOM^4^ and 14^1^ for OM^5^ |
| Gaarder **[14]** | 9.3^1^ |
| Crawford **[15]** | Nr |
| Siriratsivawong **[16]** | 17.2^1^ for OM, 12.4^1^ for sNOM^6^, 20.7^1^ for fNOM^7^ |
| Harbrecht **[17]** | Nr |
| Duchesne **[18]** | 16.1^1^ for group 1 and 14.1^1^ for group 2 |
| Bowman **[19]** | Nr |
| Jim **[20]** | 5.6^1^ for NOM, 9.67^1^ for OM ^8^and 8.53^1^ for dOM ^9^ |
| Scappellato **[21]** | Nr |
| Velmahos **[22]** | 13^1^±18SD |
| Costa **[1]** | Nr |
| Malhotra **[23]** | Nr |
| Bruce **[24]** | Nr |
| Claridge **[25]** | 6.6^10^±1.2SD for OM and 7.4^10^±0.7SD for NOM |

^1^mean

^2^Standard deviation

^3^not reported

^4^non operative management

^5^operative management

^6^ successful NOM

^7^failure NOM

^8^ early OM

^9^ delayed OM

^10^median
