# Supplementary material for: Is non-operative management safe and effective for all splenic blunt trauma? A systematic review
Source: Crit Care. 2013 Sep 3;17(5):R185. doi: 10.1186/cc12868 (PMC4056798; doi:10.1186/cc12868)
Supplement: Additional file 11 — Table S11. Hospital stay after blunt splenic trauma: NOM vs OM. [file cc12868-S11.DOCX]

Table 12:Morbidity according to Dindo-Clavien in splenic traumatic lesions: NOM vs. OM.

|  | | | | Morbidity according to Dindo-Clavien classification | | | | |
| --- | --- | --- | --- | --- | --- | --- | --- | --- |
| Study | Treatment./n° of patients | AAST^1^ | n° of patients | I | II | III | IV | V |
| Wahl [10] | OM^2^  36 | I | nr^3^ | nr | nr | nr | nr | nr |
|  |  | II | nr | nr | nr | nr | nr | nr |
|  |  | III | nr | nr | nr | nr | nr | nr |
|  |  | IV | nr | nr | nr | nr | nr | nr |
|  |  | V | nr | nr | nr | nr | nr | nr |
|  | NOM^4^  238 | I | nr | nr | nr | nr | nr | nr |
|  |  | II | nr | nr | nr | nr | nr | nr |
|  |  | III | nr | nr | nr | nr | nr | nr |
|  |  | IV | nr | nr | nr | nr | nr | nr |
|  |  | V | nr | nr | nr | nr | nr | nr |
| Duchesne [18] | OM  78 | I | 5 | 0 | 0 | 0 | 0 | 0 |
|  |  | II | 7 | 0 | 0 | 0 | 0 | 0 |
|  |  | III | 21 | 0 | 0 | 0 | 3 | 4 |
|  |  | IV | 29 | 0 | 0 | 0 | 2 | 6 |
|  |  | V | 16 | 0 | 0 | 0 | 3 | 4 |
|  | NOM  76 | I | 10 | 0 | 0 | 0 | 1 | 0 |
|  |  | II | 16 | 0 | 0 | 0 | 3 | 0 |
|  |  | III | 25 | 0 | 0 | 0 | 7 | 5 |
|  |  | IV | 19 | 0 | 0 | 0 | 11 | 4 |
|  |  | V | 6 | 0 | 0 | 0 | 4 | 2 |
| Velmahos [22] | OM  164 | I | 0 | 0 | 0 | 0 | 0 | 0 |
|  |  | II | 0 | 0 | 0 | 0 | 0 | 0 |
|  |  | III | 0 | 0 | 0 | 0 | 0 | 0 |
|  |  | IV | 121 | nr | nr | nr | nr | nr |
|  |  | V | 43 | nr | nr | nr | nr | nr |
|  | NOM  224 | I | 0 | 0 | 0 | 0 | 0 | 0 |
|  |  | II | 0 | 0 | 0 | 0 | 0 | 0 |
|  |  | III | 0 | 0 | 0 | 0 | 0 | 0 |
|  |  | IV | 193 | nr | nr | nr | nr | nr |
|  |  | V | 31 | nr | nr | nr | nr | nr |
| Total | OM  278 | I | 5 | 0 | 0 | 0 | 0 | 0 |
|  |  | II | 7 | 0 | 0 | 0 | 0 | 0 |
|  |  | III | 21 | 0 | 0 | 0 | 3 | 4 |
|  |  | IV | 150 | 0 | 0 | 0 | 2 | 6 |
|  |  | V | 59 | 0 | 0 | 0 | 3 | 4 |
|  | NOM  538 | I | 10 | 0 | 0 | 0 | 1 | 0 |
|  |  | II | 16 | 0 | 0 | 0 | 3 | 0 |
|  |  | III | 25 | 0 | 0 | 0 | 7 | 5 |
|  |  | IV | 212 | 0 | 0 | 0 | 11 | 4 |
|  |  | V | 37 | 0 | 0 | 0 | 4 | 2 |

^1^ classification of the American Association for the Surgery of Trauma

^2^operative management

^3^not reported

^4^non operative management
